# Editorial: Proteomics of plant development and hormonal responses, volume II

**DOI:** 10.3389/fpls.2023.1340170

**Published:** 2023-11-28

**Authors:** Alexandra M.E. Jones, Ive De Smet

**Affiliations:** ^1^ School of Life Sciences, University of Warwick, Coventry, United Kingdom; ^2^ Department of Plant Biotechnology and Bioinformatics, Ghent University, Ghent, Belgium; ^3^ VIB Center for Plant Systems Biology, Ghent, Belgium

**Keywords:** watermelon, arabidopsis, soybean, proteome, phosphoproteome, transcriptome

Plant development relies for a large part on internal cues, including phytohormones (Depuydt and Hardtke, 2011; Durbak et al., 2012). Several phytohormones directly impact protein levels or activity, for example, through post-translational modifications or protein degradation (Dharmasiri and Estelle, 2004; Kim and Russinova, 2020). Proteomics is a powerful research tool to decipher molecular responses during a plant’s life and to increase our understanding of cellular processes beyond transcriptional changes. In this Research Topic, we focus on the application of proteomics and mass spectrometry to plant developmental and hormonal responses.

Fruit ripening is modulated by many factors, including phytohormones. Yu et al. generated transcriptomic and proteomic data for fruit development and ripening in watermelon. In addition to the valuable resource for watermelon research, their analyses of the transcriptome and proteome revealed that mRNA and protein levels were poorly correlated.

Lardon et al. probed dynamic protein phosphorylation during *de novo* shoot organogenesis in *Arabidopsis thaliana* in the context of histidine kinases and cytokinin signaling. The deregulation of protein phosphorylation in Ser, Thr or Tyr provided insight in novel regeneration determinants.

There are also interactions with the environment that impact plant development. Salt stress is a major environmental factor limiting plant growth and development. Feng et al. generated *Arabidopsis thaliana* transcriptome and proteome data associated with the activity of the brassinosteroid-regulated transcription factor BES1. The interplay between salt stress tolerance, microbes and the plant proteome is explored by Ilangumaran et al. through shotgun proteomics, who demonstrated that proteins related to soybean growth and stress tolerance were modulated upon bacterial inoculation. Their findings suggest that plant growth promoting rhizobacteria regulate the soybean leaf proteome through multiple signaling pathways to improve plant growth upon salt stress.

In this Research Topic, mass spectrometry-driven approaches haven been used successfully to unravel signalling pathways in diverse processes in diverse species. This lays the foundation for more in depth analyses in the context of plant growth and development associated with the biotic and abiotic environment and facilitates the identification of key signaling components.

## Author contributions

AJ: Writing – review & editing. ID: Writing – original draft, Writing – review & editing.
